# Habitual Smoking and Perinatal Outcomes in Japan

**DOI:** 10.7759/cureus.22426

**Published:** 2022-02-21

**Authors:** Shunji Suzuki

**Affiliations:** 1 Obstetrics and Gynecology, Nippon Medical School, Tokyo, JPN

**Keywords:** smoking cessation, japanese women, perinatal outcomes, pregnancy, smoking

## Abstract

Introduction: Smoking during pregnancy has been reported to increase the perinatal risk. We investigated the perinatal outcomes in Japanese pregnant women with habitual smoking at pre-pregnancy and during pregnancy who delivered at our institute in Japan.

Methods: We reviewed the obstetric records of 9280 Japanese women with singleton delivery at ≥22 weeks of gestation at our institute between 2014 and 2019. On admission for delivery, a routine face-to-face interview was conducted by our midwives to ask about smoking during pregnancy.

Results: Of the total 9280 pregnant Japanese women, 532 (5.7%) had smoked before pregnancy. Of these, 272 (51.5%) quitted smoking after being diagnosed with pregnancy, while 258 (49.5%) continued to smoke during pregnancy and were diagnosed with habitual smoking during pregnancy. There was no significant relation between habitual smoking at pre-pregnancy and the perinatal adverse outcomes. However, habitual smoking during pregnancy was associated with the adverse perinatal outcomes such as the increased incidence of placental abruption (adjusted odds ratio [OR] 2.38, p = 0.04) and light-for-gestational-age neonate (adjusted OR 1.72, p = 0.03) on multiple logistic regression analyses.

Conclusion: In this study, smoking was found to be associated with the adverse perinatal outcomes. The current results support the importance of smoking cessation during pregnancy.

## Introduction

Smoking during pregnancy has been reported to increase the risk of abortion, placental abruption, stillbirth, preterm labor and congenital anomalies [1]. In addition, the relation between maternal smoking and fetal growth restriction has seemed to be well investigated [1,2]. Smoking has been observed to be associated with the abnormal development of placental vascularization leading to placental insufficiency reducing the feto-maternal circulation and in early pregnancy has been suggested to be associated with villus hypoxia resulting in angiogenesis and apoptosis [3].

In 2021, smoking during pregnancy was reported to be identified as one of the main risk factors for cerebral palsy following placental abruption in Japan [4]. Among the pregnant women who developed placental abruption registered in the Japan Obstetric Compensation System for Cerebral Palsy (JOCSC), the rate of smoking during pregnancy was reported to be 9.7% [5]. However, there have been few investigations of widespread examinations of habitual smoking during pregnancy and perinatal outcomes in Japanese women [4,6]. Therefore, we investigated the perinatal outcomes in women diagnosed with habitual smoking during pregnancy and were managed at our institute, which is one of the main perinatal centers in Tokyo, Japan.

## Materials and methods

The protocol of this study was approved by the Ethics Committee of the Japanese Red Cross Katsushika Maternity Hospital. Informed consent for the publication of this study was obtained from the patients.

We reviewed the obstetric records of 9280 Japanese women who delivered singleton neonates at ≥22 weeks of gestation at our institute between 2014 and 2019. Our institute is one of main perinatal centers in Tokyo, Japan (with about 1800-2000 deliveries per year). On admission for delivery, a routine face-to-face interview was conducted by our midwives to ask about smoking during pregnancy. When a pregnant woman smoked continuously (daily or occasionally, at least one cigarette per day) during pregnancy, we diagnosed her with habitual smoking during pregnancy based on the definition by the Japan Ministry of Health, Labour and Welfare [7]. To examine the relation between habitual smoking at pre-pregnancy and the adverse perinatal outcomes, the subjects were divided into three groups: (1) those with habitual smoking during pregnancy (i.e., those who did not quit smoking when they got pregnant), (2) those with habitual smoking at pre-pregnancy (i.e., those who quitted smoking with the diagnosis of pregnancy), and (3) non-smoking, as control. From the patient charts, demographic information and obstetric characteristics, such as the maternal age, parity, perinatal complications, gestational age at delivery, delivery modes, birth weight of the neonate, Apgar score at one and five minutes, and umbilical artery pH, were extracted.

Data are presented as the number (percentage) or mean ± standard deviation (SD). The chi-square test was used for categorical variables, while the Student’s t-test was used for continuous variables for statistical analyses. Odds ratios (ORs) and 95% confidence intervals (CIs) were calculated. Differences with p < 0.05 were considered significant. Variables used in the multivariate model were those that had shown a marginally significant (p < 0.05) relation with the perinatal outcomes in univariate analysis.

## Results

Of the 9280 Japanese women, 532 (5.7%) had smoked before pregnancy. Of these, 272 quitted smoking after being diagnosed with pregnancy (51.5% with habitual smoking at pre-pregnancy; 3.0% of the total), while 258 continued to smoke during pregnancy and were diagnosed with habitual smoking during pregnancy (49.5%; 2.8% of the total). According to the interviews, most from the latter group seemed to smoke 5-10 cigarettes per day.

Table 1 shows the clinical characteristics of Japanese women with deliveries at ≥22 weeks of gestation with and without habitual smoking. In univariate analysis, habitual smoking was associated with a low maternal age, nulliparity and habitual alcohol consumption during pregnancy.

**Table 1:** Clinical characteristics of Japanese women who delivered at ≥22 weeks of gestation with and without habitual smoking *Versus control group (non-smokers)

Characteristics	Control (n=8748)	Habitual smoking at pre-pregnancy (n=274)	p-value*	Habitual smoking during pregnancy (n=258)	p-value*
Maternal age					
Average (years)	32.7±5.5	31.9±6.8	<0.01	30.8±7.4	<0.01
<20 years	95 (1.1%)	6 (2.1%)	0.09	12 (4.7%)	<0.01
≥35 years	3381 (38.6%)	109 (39.8)	0.71	105 (40.7%)	0.52
Nulliparity	4431 (50.7%)	132 (48.2%)	0.44	93 (36.0%)	<0.01
Habitual drinking	24 (0.2%)	16 (5.9%)	<0.01	40 (15.5%)	<0.01
Maternal height (cm)	158.2±6.5	158.3±5.8	0.52	158.4±5.6	0.21
Maternal weight (kg)					
At pre-pregnancy	53.3±8.9	53.6±9.0	0.10	53.6±9.5	0.11
At delivery	63.7±9.4	64.0±9.5	0.11	64.1±8.8	0.07

Table 2 shows the perinatal outcomes of the Japanese pregnant women with and without habitual smoking. There was no significant relation between habitual smoking at pre-pregnancy and the perinatal adverse outcomes. In univariate analysis, habitual smoking during pregnancy was more likely with the presence of hypertensive disorders (crude OR 1.63, 95% CI 1.1-2.5, p = 0.02), placental abruption (crude OR 2.69, 95% CI 1.4-5.3, p = 0.01), preterm delivery (crude OR 2.22, 95% CI 1.4-3.4, p < 0.01), low birth weight (crude OR 1.69, 95% CI 1.2-2.3, p = 0.02) and light-for-gestational-age neonate (crude OR 2.08, 95% CI 1.5-3.0, p < 0.01).

**Table 2:** Perinatal outcomes of Japanese women who delivered at ≥22 weeks of gestation with and without habitual smoking *Versus control group (non-smokers)

Characteristics	Control (n=8748)	Habitual smoking at pre-pregnancy (n=274)	p-value*	Habitual smoking during pregnancy (n=258)	p-value*
Hypertensive disorders	523 (6.0%)	19 (6.9%)	0.48	24 (9.3%)	0.02
Placental abruption	118 (1.3%)	3 (1.1%)	0.72	9 (3.5%)	0.01
Gestational age at delivery					
Average (weeks)	38.6±2.2	38.4±2.0	0.08	38.5±2.0	0.26
<37 weeks	386 (4.4%)	14 (5.1%)	0.58	24 (9.3%)	<0.01
Cesarean delivery	1731 (19.8%)	51 (18.6%)	0.63	49 (19.0%)	0.75
Neonatal birth weight					
Average (g)	2948±511	2929±459	0.66	2855±488	0.03
<2500 g	1157 (13.2%)	38 (13.9)	0.76	52 (20.5%)	0.02
Light for gestational age	672 (7.7%)	22 (8.0%)	0.83	38 (14.9%)	<0.01
Intrauterine fetal death	20 (0.2%)	1 (0.4%)	0.65	2 (0.8%)	0.08
Apgar score					
<7 at 1 minute	140 (1.6%)	2 (0.8%)	0.25	3 (1.1%)	0.58
<7 at 5 minutes	17 (0.2%)	1 (0.4%)	0.06	0 (0%)	0.48
Umbilical artery pH <7.0	8 (0.1%)	1 (0.4%)	0.16	1 (0.4%)	0.14
Maternal blood loss ≥1000 ml	438 (5.0%)	19 (6.9%)	0.15	18 (7.0%)	0.16

In multiple logistic regression analyses, habitual smoking was found to be associated with placental abruption (adjusted OR 2.38, 95% CI 1.0-4.5, p = 0.04) and light-for-gestational-age neonate (adjusted OR 1.72, 95% CI 1.0-2.8, p = 0.03) as shown in Table 3.

**Table 3:** Association between habitual smoking during pregnancy and perinatal outcomes in Japanese women who delivered at ≥22 weeks of gestation

	p-value	Adjusted odds ratio	95% Confidence interval
Hypertensive disorders	0.06	1.63	0.91-2.4
Placental abruption	0.04	2.38	1.00-4.52
Gestational age at delivery			
<37 weeks	0.09	1.58	0.88-3.0
Neonatal birth weight			
Average (g)	0.08	-	-
<2500 g	0.07	1.40	0.74-2.2
Light for gestational age	0.03	1.72	1.03-2.81

## Discussion

The present study found that maternal habitual smoking in pregnancy was associated with adverse perinatal outcomes such as the increased incidence of placental abruption and light-for-gestational-age neonate as previously reported [1-4,6]. The characteristic abnormalities caused by smoking have been reported to provide additional insights into the genes and processes important for the formation of the fetal-maternal interface associated with the adverse perinatal consequences [3]. Recently, the variation in frequencies of placental abruption across countries has been suggested to be associated with differences in the distribution of risk factors, especially smoking, and our observations may help guide policy to reduce the incidence of placental abruption [8]. The current results also support some other previous studies conducted in Japan [9,10].

Although pregnancy is probably an event that mostly motivates females to try quitting smoking, little is known about the appropriate timing of their quit attempts [11]. Therefore, women should be encouraged to quit smoking before becoming pregnant [12]. However, the current study indicated the possibility to prevent the deterioration of the perinatal outcomes at ≥22 weeks of gestation even if smoking cessation is started after becoming pregnant.

Placental dysfunction has been investigated to be a well-established cause of fetal growth restriction, and maternal smoking has been reported to be positively associated with growth restriction associated with the placental circulatory dysfunction. Adverse intrauterine conditions beginning in early pregnancy that may cause fetal malnutrition, such as smoking, have been suggested to result in the pathogenesis of light-for-gestational-age neonate as shown by the current results [13,14].

In this study, there was no significant relation between habitual smoking at pre-pregnancy and the perinatal adverse outcomes. In Japan, the proportion of people who smoke habitually has decreased significantly for both men and women over the last decade [15]. Smoking cessation during pregnancy has been suggested to reduce the risk of adverse perinatal outcomes [1,4]. The perinatal risks due to smoking have been found to increase with the increase in the number of cigarettes; however, women who stopped smoking during pregnancy seemed to be at the lower levels for the pathologies of perinatal complications [1,4]. The previous reports and the current results support the importance of the education of pregnant women for smoking cessation and the influence of smoking during pregnancy.

According to the survey of the Japan Ministry of Health, Labor and Welfare in 2019, the proportion of women who smoked habitually was 7.6%, which was 7.4% among those in their 30s [15]. In addition, in this study, habitual smoking seemed to be associated with low maternal age and habitual alcohol consumption during pregnancy. These trends seem to be similar to the survey results [15]. Perhaps due to various smoking cessation instructions, the smoking rate in pregnant women in Japan seemed to be lower than that for non-pregnant women of the same generation, as shown by the current results [16]. According to the current study, about half of pregnant women quitted smoking after finding out that they were pregnant. This frequency may be similar to or lower than that found in other countries due to the smoking rates varying widely from country to country [8,9]. In addition, there may be regional differences among Japanese pregnant women as well [4,15]. Otherwise, it may be the result of many women quitting smoking before becoming pregnant in Japan [4,15].

Although the results seemed to be consistent with the evidence previously reported, this study was not without limitations [1,7]. Firstly, because of the small sample size of the study, we could not examine the other serious problems associated with smoking such as birth defects. Secondly, we could not examine the risk of sudden infant death syndrome because of the short term of the study. Thirdly, we did not consider women's partners' habitual smoking practices or work environments. While carrying out the current analyses, we believed in interviews with the pregnant women regarding the smoking situation; however, we cannot deny the fact that there may be doubts about their reliability.

In addition, based on our previous report, we should keep in mind that pregnant women with habitual smoking often show habitual alcohol consumption associated with the adverse perinatal outcomes including fetal neurodevelopmental abnormalities [4,17,18]. In the subjects of this study, the rate of habitual drinking in the habitual smoking group was significantly higher than that in the control group according to the interview with them at the time of delivery (15.5% vs. 0.2%, p < 0.01; Kato M, Suzuki S: Habitual Alcohol Consumption During Pregnancy and Perinatal Outcomes, 50th Congress of Japan Society of Perinatal and Neonatal Medicine, Fukuoka, Japan, July 10, 2015). However, in this study, alcohol consumption is difficult to be considered as one of the confounding factors accurately either. We understand that the main restriction of this study is the study design because retrospective data may be defective in terms of data quality; hence, pregnant women with habitual smoking practices should be managed considering various perinatal risks.

## Conclusions

In conclusion, habitual smoking in pregnancy seemed to be associated with adverse perinatal outcomes such as increased incidence of placental abruption and light-for-gestational-age neonate. However, it seemed to be possible to improve the perinatal outcomes even if smoking cessation is started after becoming pregnant. These findings support the importance of smoking cessation during pregnancy.

## References

[1] Polańska K, Hanke W (2004). Effect of smoking during pregnancy on maternal condition and birth outcome--overview of epidemiologic studies. (Article in Polish). Przegl Epidemiol.

[2] Pintican D, Poienar AA, Strilciuc S, Mihu D (2019). Effects of maternal smoking on human placental vascularization: a systematic review. Taiwan J Obstet Gynecol.

[3] Kawashima A, Koide K, Ventura W (2014). Effects of maternal smoking on the placental expression of genes related to angiogenesis and apoptosis during the first trimester. PLoS One.

[4] Ichizuka K, Toyokawa S, Ikenoue T (2021). Risk factors for cerebral palsy in neonates due to placental abruption. J Obstet Gynaecol Res.

[5] Japan Council for Quality Health Care (2021). Japan Council for Quality Health Care. The Japan Obstetric Compensation System for Cerebral Palsy. (In Japanese). http://www.sanka-hp.jcqhc.or.jp/documents/prevention/index.html.

[6] Suzuki M, Wakayama R, Yamagata Z, Suzuki K (2022). Effect of maternal smoking during pregnancy on gestational weight gain and birthweight: a stratified analysis by pregestational weight status. Tob Induc Dis.

[7] (2021). Japan Ministry of Health, Labor and Welfare. Information about smoking and health. (In Japanese). https://www.mhlw.go.jp/stf/seisakunitsuite/bunya/kenkou_iryou/kenkou/tobacco/index.html.

[8] Ananth CV, Keyes KM, Hamilton A (2015). An international contrast of rates of placental abruption: an age-period-cohort analysis. PLoS One.

[9] Zheng W, Suzuki K, Shinohara R, Sato M, Yokomichi H, Yamagata Z (2015). Maternal smoking during pregnancy and growth in infancy: a covariance structure analysis. J Epidemiol.

[10] Terada M, Matsuda Y, Ogawa M, Matsui H, Satoh S (2013). Effects of maternal factors on birth weight in Japan. J Pregnancy.

[11] Cooper S, Orton S, Leonardi-Bee J (2017). Smoking and quit attempts during pregnancy and postpartum: a longitudinal UK cohort. BMJ Open.

[12] Mendelsohn C, Gould GS, Oncken C (2014). Management of smoking in pregnant women. Aust Fam Physician.

[13] Chew LC, Verma RP (2021). Fetal growth restriction. In StatPearls [Internet].

[14] Morales-Prieto DM, Fuentes-Zacarías P, Murrieta-Coxca JM, Gutierrez-Samudio RN, Favaro RR, Fitzgerald JS, Markert UR (2021). Smoking for two - effects of tobacco consumption on placenta. (Online ahead of print). Mol Aspects Med.

[15] (2021). Japan Ministry of Health, Labor and Welfare. National Health and Nutrition Survey. (In Japanese). http://www.health-net.or.jp/tobacco/product/pd100000.html.

[16] Minakami H, Maeda T, Fujii T (2014). Guidelines for obstetrical practice in Japan: Japan Society of Obstetrics and Gynecology (JSOG) and Japan Association of Obstetricians and Gynecologists (JAOG) 2014 edition. J Obstet Gynaecol Res.

[17] Ikeda M, Suzuki S (2015). Habitual alcohol consumption during pregnancy and perinatal outcomes. J Nippon Med Sch.

[18] O'Leary CM, Watson L, D'Antoine H, Stanley F, Bower C (2012). Heavy maternal alcohol consumption and cerebral palsy in the offspring. Dev Med Child Neurol.

